# Predicting 90-day prognosis for patients with stroke: a machine learning approach

**DOI:** 10.3389/fneur.2023.1270767

**Published:** 2023-12-07

**Authors:** Ahmad A. Abujaber, Ibraheem M. Alkhawaldeh, Yahia Imam, Abdulqadir J. Nashwan, Naveed Akhtar, Ahmed Own, Ahmad S. Tarawneh, Ahmad B. Hassanat

**Affiliations:** ^1^Nursing Department, Hamad Medical Corporation (HMC), Doha, Qatar; ^2^Faculty of Medicine, Mutah University, Al-Karak, Jordan; ^3^Neurology Section, Neuroscience Institute, Hamad Medical Corporation (HMC), Doha, Qatar; ^4^Neuroradiology Department, Neuroscience Institute, Hamad Medical Corporation (HMC), Doha, Qatar; ^5^Faculty of Information Technology, Mutah University, Al-Karak, Jordan

**Keywords:** stroke, prognosis, ischemic stroke, hemorrhagic stroke, machine learning

## Abstract

**Background:**

Stroke is a significant global health burden and ranks as the second leading cause of death worldwide.

**Objective:**

This study aims to develop and evaluate a machine learning-based predictive tool for forecasting the 90-day prognosis of stroke patients after discharge as measured by the modified Rankin Score.

**Methods:**

The study utilized data from a large national multiethnic stroke registry comprising 15,859 adult patients diagnosed with ischemic or hemorrhagic stroke. Of these, 7,452 patients satisfied the study’s inclusion criteria. Feature selection was performed using the correlation and permutation importance methods. Six classifiers, including Random Forest (RF), Classification and Regression Tree, Linear Discriminant Analysis, Support Vector Machine, and k-Nearest Neighbors, were employed for prediction.

**Results:**

The RF model demonstrated superior performance, achieving the highest accuracy (0.823) and excellent discrimination power (AUC 0.893). Notably, stroke type, hospital acquired infections, admission location, and hospital length of stay emerged as the top-ranked predictors.

**Conclusion:**

The RF model shows promise in predicting stroke prognosis, enabling personalized care plans and enhanced preventive measures for stroke patients. Prospective validation is essential to assess its real-world clinical performance and ensure successful implementation across diverse healthcare settings.

## Background

Stroke is a global health concern, recognized as the second leading cause of death and a prominent contributor to long-term disability worldwide (1). The World Health Organization estimates that annually, 13.7 million people suffer a stroke, and approximately 5.5 million succumb to death due to its complications (1). Furthermore, stroke is a primary cause of significant, persistent disability. Over half of the stroke survivors aged 65 and over experience reduced mobility due Stroke (2, 3). In Qatar, Hamad General Hospital (HGH), the country’s main tertiary hospital, offers specialized stroke services under the umbrella of the Neuroscience Institute and maintains the stroke registry. This center covers more than 90% of all stroke admissions. The registry provides a comprehensive insight into the incidence and management of stroke disease in the country. Qatar is home known for its multiethnic population with a large young able-bodied South/Southeastern Asian presence. Whereas the local Qatari population have an average incidence of stroke is 92.04 per 100,000 adult population (4). The mean age is around 64 years, and the average age for the first cerebrovascular event is approximately 63 years (4). The most common type of stroke was ischemic stroke (IS), accounting for 73.7% of cases, primarily caused by small vessel disease (4). Hypertension and diabetes were particularly common in this group, affecting 82.7 and 71.6% of patients, respectively (4). Compared to their male counterparts, Qatari females were older at the time of stroke onset, experienced higher rates of hypertension and diabetes, and had a greater likelihood of disability or death at 90 days (4). The Registry is a prospective one that captures important clinicodemographic characteristics of stroke patients and their complications and outcomes (5).

This substantial global and national burden emphasizes the urgency to optimize stroke management strategies, including the identification of robust prognostic factors that could inform therapeutic decisions and patient care pathways.

## Literature review

The research community has made substantial strides in examining the factors contributing to stroke outcomes. These encompass patient demographics, clinical characteristics, and treatment modalities, as well as the exploration of machine learning models to predict prognosis. Age, gender, and pre-stroke health status, such as the pre-admission modified Rankin Score (mRS), were frequently noted as significant factors in the prognosis of patients post-thrombectomy (6). Similarly, lifestyle habits, like smoking, were found to influence outcomes, with non-smokers more likely to have a favorable recovery (7).

Clinical indicators are another common factor in predicting stroke outcomes. Infarct volume, for instance, has been linked to clinical outcomes following IS. Smaller infarct growths, as well as better initial perfusion, have been associated with better patient outcomes (8). Likewise, post-thrombectomy National Institutes of Health Stroke Scale (NIHSS) scores and the requirement for a decompressive hemicraniectomy were identified as significant predictors of functional outcomes (6). Furthermore, stroke severity, indicated by NIHSS scores, along with alteplase treatment, was noted as significant in determining functional changes in mild IS patients from 30 to 90 days post-stroke (9).

The predictability of various scoring systems was examined, and the modified SOAR (mSOAR) score was reported to be effective in predicting post-stroke disability (10). Similarly, the impact of factors such as age, stroke history, heart rate, and TOAST classification on the prognosis of transient ischemic attack (TIA) or minor stroke patients was discussed, and they were integrated into machine learning models for predictive purposes (11).

The application of machine learning algorithms for predicting stroke outcomes is promising. These algorithms were found to have comparable, if not superior, performance in predicting the 90-day prognosis of TIA and minor stroke patients compared to traditional logistic regression models. Similarly, explainable machine learning methodologies have been developed to predict functional outcomes at discharge, showing high levels of accuracy (11).

Predicting outcomes post-stroke is paramount for clinical planning and patient care. A common measure of disability and independence in patients after suffering a stroke is the mRS (11, 12). An analysis of acute IS patients found significant changes in mRS scores from 30 to 90 days post-discharge (11). The mRS score at discharge and non-home discharge disposition were deemed good individual predictors of the 90-day mRS score, providing a tool for assessing likely patient outcomes (11). Accurate prediction of mRS scores can guide patient expectations and clinical trial analyses. A model developed using data from multi-center prospective studies predicted the 90-day mRS score based on variables available during the stroke hospitalization (13). This model found age and NIHSS score at discharge as significant independent predictors of the 90-day mRS, with an accuracy of 78% in predicting the mRS score within one point in the validation cohort (13).

Further to this, machine learning models have been shown to offer promising results in predicting patient outcomes. With the ability to analyze a vast amount of clinical, laboratory, and imaging data, these models can provide nuanced insight. In one study, different machine learning algorithms, including XGBoost, LightGBM, CatBoost, and Random Forest (RF), were used to predict short- and medium-term functional outcomes in acute IS patients (14, 15). The LightGBM and Random Forest algorithms demonstrated the highest predictive power for functional outcomes (14).

Despite a mild onset, patients with IS can still exhibit substantial disability rates at 90 days, often due to early neurological worsening (16). A prospective cohort study found that early worsening and acute infarct growth from baseline to 5 days were more common among those with poor outcomes (16). On the other hand, studies on thrombectomy in late time windows have reported improved patient outcomes. In the DEFUSE 3 study, patients who exhibited rapid neurological improvement (RNI) 24 h after thrombectomy were more likely to have a favorable clinical outcome (17). RNI was associated with a favorable shift in the mRS at day 90 and lower rates of mortality (17).

Stroke outcome prediction is a multifaceted process that integrates a range of factors, from demographic and clinical characteristics to the incorporation of machine learning models. These advancements have the potential to refine the prognostic process, enabling personalized therapeutic strategies, improving patient outcomes, and mitigating the global burden of stroke. Therefore, this study aims at designing a machine learning based model to help predict the stroke patient’s prognosis 90 days post discharge using mRS.

## Materials and methods

**Figure 1:**
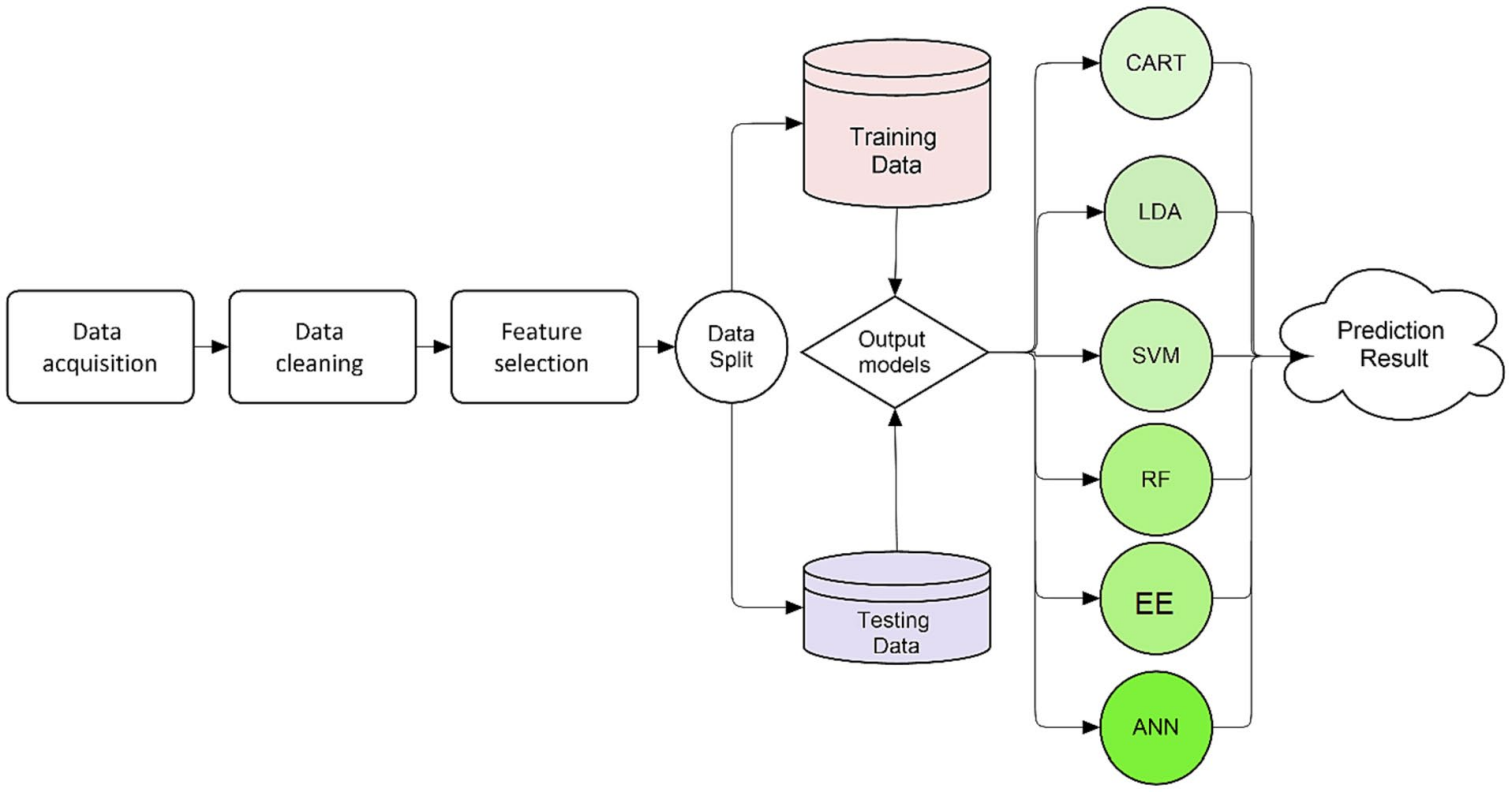
Summary of study’s methodology.

The study received approval from the institutional research board (IRB) of Hamad Medical Corporation, Qatar, with reference MRC-01-22-594. The study methodology followed a specific sequence of steps as summarized in Figure 1.

### Data collection

Data were collected from the Stroke Registry of Hamad General Hospital (HGH), covering the period from January 2014 to July 2022. The dataset includes all adults aged 18 years and above, who were admitted to HGH with a primary diagnosis of stroke, comprising cases of IS, transient ischemic attack (TIA), hemorrhagic stroke (ICH), and stroke mimics. In total, 15,859 patients sought specialized stroke care at the hospital since the establishment of the stroke registry in Qatar.

### Baseline variables

The extracted variables covered diverse aspects of the patients, including demographic information, ethnicity, stroke risk factors, known comorbidities, admission location, hospitalization outcomes [i.e., length of stay (LOS) and hospital-acquired infections like pneumonia and urinary tract infection (UTI)], mortality, and stroke severity. The severity of stroke at admission was classified into five categories using the National Institute of Health Stroke Score (NIHSS) (18, 19). At the time of admission, the mRS was collected, which captures the patient’s pre-stroke condition as reported by family members, graded on a 0–6 scale (11). IS etiology was determined using the Trial of Org 10,172 in Acute Stroke Treatment (TOAST) classification (20). A stroke type variable was created by combining the five TOAST categories under IS, with ICH forming another category, providing a comparative perspective between the two stroke types. To identify Body Mass Index (BMI) categories, the CDC’s 5-class definition for adult overweight and obesity was adopted (21).

Regarding ethnicity, patients were classified into five groups based on their declared nationality: Qatari, Middle East and North Africa (MENA) region, South Asia region, South East Asia region (as defined by the United Nations geo-scheme), and all other nationalities categorized as “other” (22, 23). Notably, the Qatari patients were placed in a separate category to facilitate a meaningful comparative perspective, considering the unique demographic structure of the country, where the majority of the population comprises expatriates (4, 24). This approach has been consistently employed in previous publications studying stroke in Qatar (5, 22). All included risk factors, such as comorbidities and smoking history, were reliably confirmed during the patient’s hospital stay and verified by the stroke registry personnel by accessing the patient’s electronic medical records. Table 1 summarizes the data.

**Table 1:** Statistical characteristics of the collected stroke dataset.

Variable	Feature	Favorable (mRS > 2)	Unfavorable (mRS >2)	Total
Age	<mean (54.32)	1985	1,512	3,497
	≥mean (54.32)	2,774	1,181	3,955
	Mean ± SD (54.32 ± 13.7)-IQR 18			
Sex	1: Male	3,944	1991	5,935
	2: Female	815	702	1,517
Ethnicity	1: Qatari	700	664	1,364
	2: MENA	895	511	1,406
	3: South Asian	2,456	1,171	3,627
	4: South-East Asian	488	250	738
	5: Other	220	97	317
Modified Rankin Score (mRS) pre-stroke onset	0: No symptoms	4,500	1913	6,413
	1: No significant disability	72	40	112
	2: Slight disability	128	128	256
	3: Moderate disability	44	313	357
	4: Moderate–Severe disability	11	160	171
	5: Severe disability	4	139	143
NIHSS at admission	1: No stroke	927	166	1,093
	2: Minor Stroke	2,596	577	3,173
	3: Moderate Stroke	1,087	1,045	2,132
	4: Moderate to Severe Stroke	85	377	462
	5: Severe Stroke	64	528	592
Body Mass Index (BMI)	0: Missing	346	47	393
	1: Underweight	168	139	307
	2: Normal weight	1,281	745	2026
	3: Overweight	1820	1,059	2,879
	4: Obese	816	454	1,270
	5: Extremely Obese	328	249	577
Diabetes Mellitus (DM)	0: No	2,348	1,185	3,533
	1: Yes	2,411	1,508	3,919
Hypertension (HTN)	0: No	1,369	597	1966
	1: Yes	3,390	2096	5,486
Dyslipidemia	0: No	2,564	1,593	4,157
	1: Yes	2,195	1,100	3,295
Prior stroke	0: No	4,338	2,280	6,618
	1: Yes	421	413	834
Atrial Fibrillation (AF)	0: No	4,602	2,451	7,053
	1: Yes	157	242	399
Coronary Artery Disease (CAD)	0: No	4,286	2,306	6,592
	1: Yes	473	387	860
Congestive Heart Failure (CHF)	0: No	4,742	2,654	7,396
	1: Yes	17	39	56
Tobacco use	0: No	3,560	2,312	5,872
	1: Yes	1,199	381	1,580
Hospital Length of Stay – LOS (days)	< mean (6.56)	1,407	693	2,101
	≥ mean (6.56)	1,286	4,066	5,351
	Mean ± SD (6.56 ± 9)-IQR 4.7			
Admission location	1: Stroke Unit	2,289	1,167	3,456
	2: ICU	203	716	919
	3: Other	2,267	810	3,077
Hospital acquired Pneumonia	0: No	4,712	2,270	6,982
	1: Yes	47	423	470
Hospital acquired Urinary Tract Infection (UTI)	0: No	4,725	2,442	7,167
	1: Yes	34	251	285
Stroke type	1: Ischemic Stroke (IS)	4,244	1951	6,195
	2: Hemorrhagic Stroke (ICH)	515	742	1,257
90-day mRS	0: Favorable (≤2) 1: Unfavorable (>2)	4,759	2,693	7,452

### Outcome variable

The mRS, collected at the 90-day post-discharge follow-up visit, was simplified into a binary variable. An mRS score of ≤2 was categorized as favorable, indicating a good prognosis while mRS score > 2 was categorized as unfavorable, representing a poor prognosis (4, 11, 25).

### Inclusion/exclusion criteria

**Figure 2:**
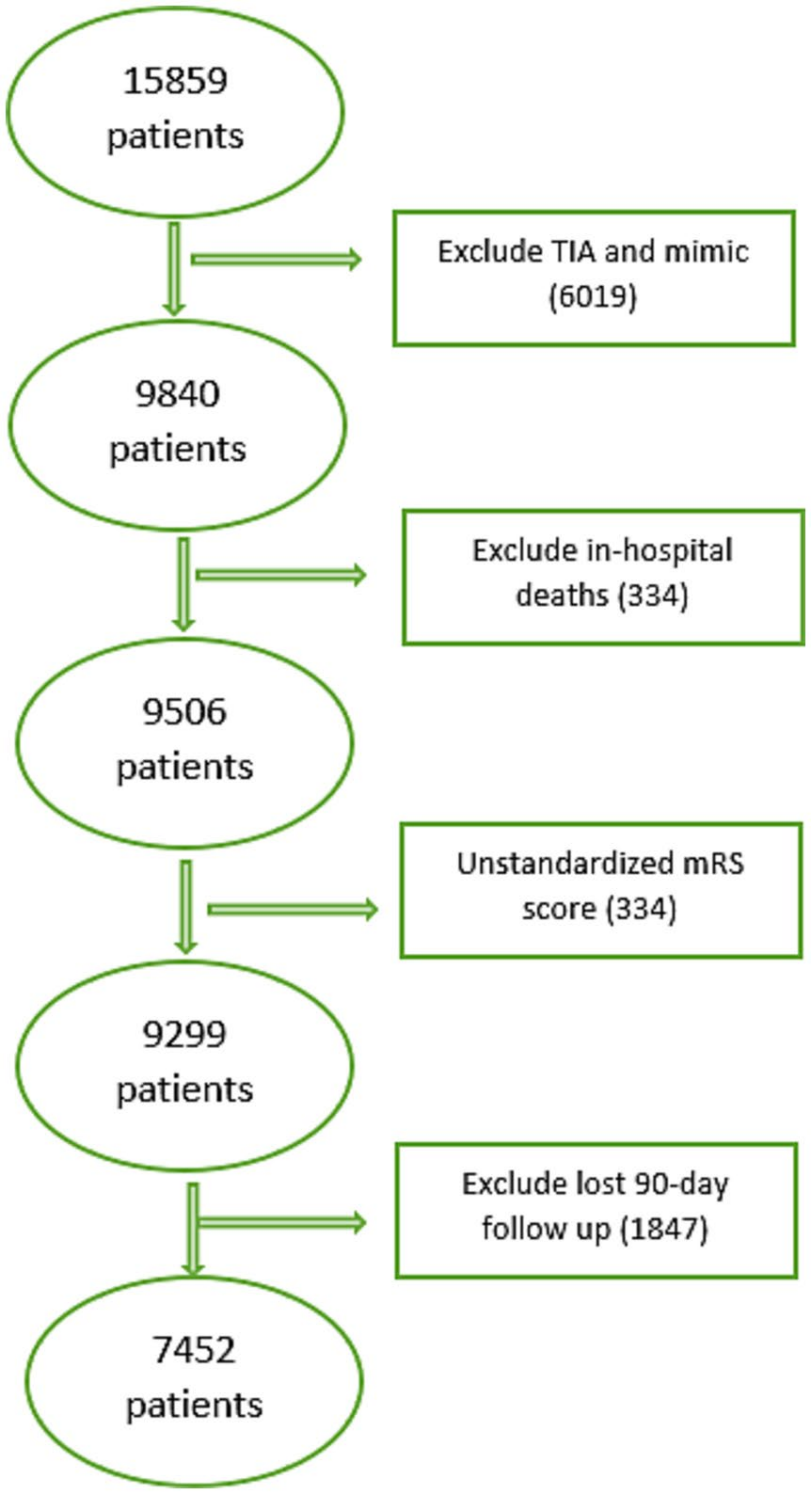
Data inclusion/exclusion process.

From initial 15,859 patients, 9,840 adults (≥18) diagnosed with IS or ICH were included. Excluded: TIA and mimic cases (6,019), in-hospital deaths (334), unstandardized (0–6) 90-day mRS score (207) and missed 90-day follow-up (1847). Finally, 7,452 patients were included in the study. See Figure 2 for details.

### Data cleaning and preprocessing

Out of the 19 variables included in the study, 18 had no missing values. However, Body Mass Index had missing values in 393 records (5%). Addressing data missingness in predictive models is crucial, and various approaches exist, such as eliminating incomplete records or imputing the missing data (26, 27). In this study, the missing values were substituted with a value of (zero). It is worth noting that, as stated by Markey et al. (28), substituting a value of zero for missing values in Body Mass Index is not recommended. However, this substitution was done merely to compare the performance of different classifiers in order to find the most effective alternative for our prediction system. The inclusion of this variable was assessed based on the results of the feature selection methods, and the whole feature is removed as can be seen from the feature selection analysis.

### Feature selection

**Figure 3:**
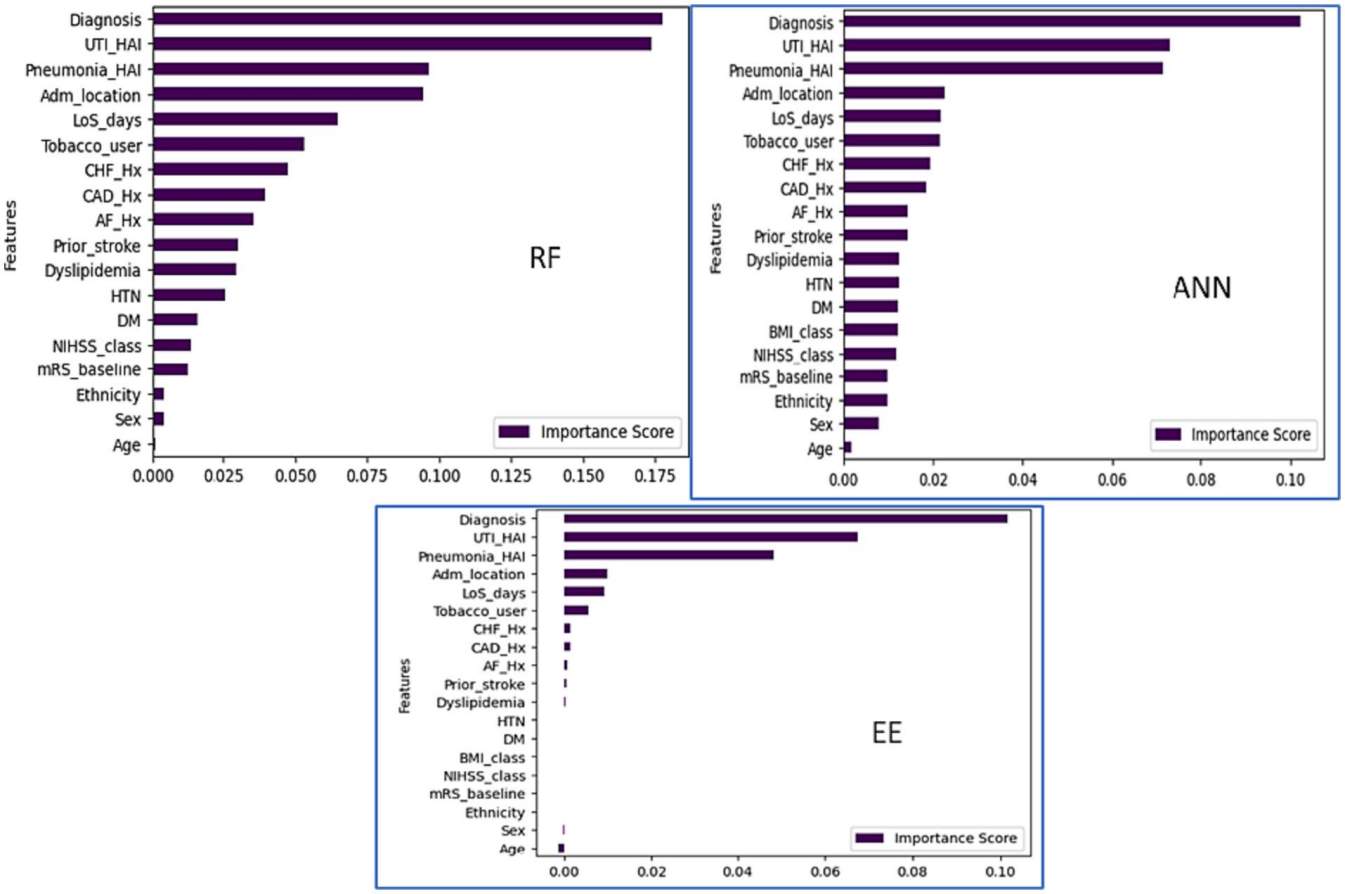
Feature importance (RF, ANN and EE classifiers).

This study used feature correlation (29) and Permutation importance (30) with RF, Easy Ensemble (EE), and Artificial Neural Network (ANN) for feature selection. Features were categorized based on correlation coefficients: weak, moderate, and strong (31, 32). Easy Ensemble identified 13 out of 19 variable (69%) and ranked them based on importance while and ANN and RF ranked all the variables based on importance (Figure 3). Finally, cross-analysis of the best features across the four approaches (EE, RF, ANN, and correlation) was done to assess the consistency and reliability of the features chosen across different methods.

### Trained models

Six classifiers were employed as follows and the training and testing process involves 5-fold cross-validation.

- Classification and Regression Tree (CART) uses decision trees to recursively split data based on feature values (33). Parameters: minimum split = 20, maximum depth = 6, complexity parameter = 0.01.
- Linear Discriminant Analysis (LDA) focuses on linear decision boundaries, maximizing separability through linear transformations (34). Parameters: prior probabilities = NULL, shrinkage coefficient = 1.
- Support Vector Machine (SVM) constructs a hyperplane with support vectors to separate classes, maximizing data point margins (35). Parameters: kernel function = “radial,” gamma = 0.2, cost: 1.
- Random Forest (RF) combines decision trees trained on random data subsets, aggregating predictions (36). Parameters: number of trees = 500, number of features = square root of all = 4, node size = 100.
- Artificial Neural Network (ANN) mimics biological neural networks, processing information through interconnected nodes with activation functions (37). Parameters: size = one hidden layer with 5 nodes, maximum number of iterations = 100, activation function = “logistic.”
- k-Nearest Neighbors (KNN) assigns a data point to the majority class among its k nearest neighbors in the feature space (38). Parameters: k = 5, distance function = “euclidean.”
- Naïve Bayes calculates the probability of each class given the values of the features independently (39). Parameters: Laplace smoothing parameter (default = 1).
- AdaBoost combines the predictions multiple weak learners (decision trees). Parameters: The type of base learner = “rpart,” the number of base learners =100, the learning rate = 0.5.
- Easy Ensemble (EE) trains multiple base learners (decision trees) on different subsets of the training data successively. It uses a weighted sampling strategy to select a portion of the training data at each iteration. Weights are assigned to training samples based on their difficulty, with heavier weights assigned to more difficult examples (40). Parameters: The number of base learners = 100, maximum depth = 3, weighting = “balanced,” sampling strategy = “random,” and learning rate = 0.5.

### Data imbalance, encoding and performance evaluation

Utilizing 5-fold validation, the study determined the best performing algorithm based on average accuracy. However, the dataset is class-imbalanced, with unfavorable mRS accounting for 36% compared to 64% favorable. To assess classifiers’ true efficacy despite class imbalance, F1-score and area under the curve (AUC) were used (41). Both factorized and one-hot encoding methods were tested to determine which method enhances the learning process and improve data representation and understanding (32). Then, a final experiment was conducted to identify the best performing algorithm using the superior encoding method.

## Results

Table 1 presents the study population’s characteristics, with an average age of 54.32 ± 13.7 years. Approximately 80% of the participants are male, and 14% were admitted with NIHSS >16. IS was diagnosed in 83% of patients, and the average LOS was 6.56 ± 9 days. At the 90-day follow-up, 36% of patients reported an unfavorable mRS score.

**Figure 4:**
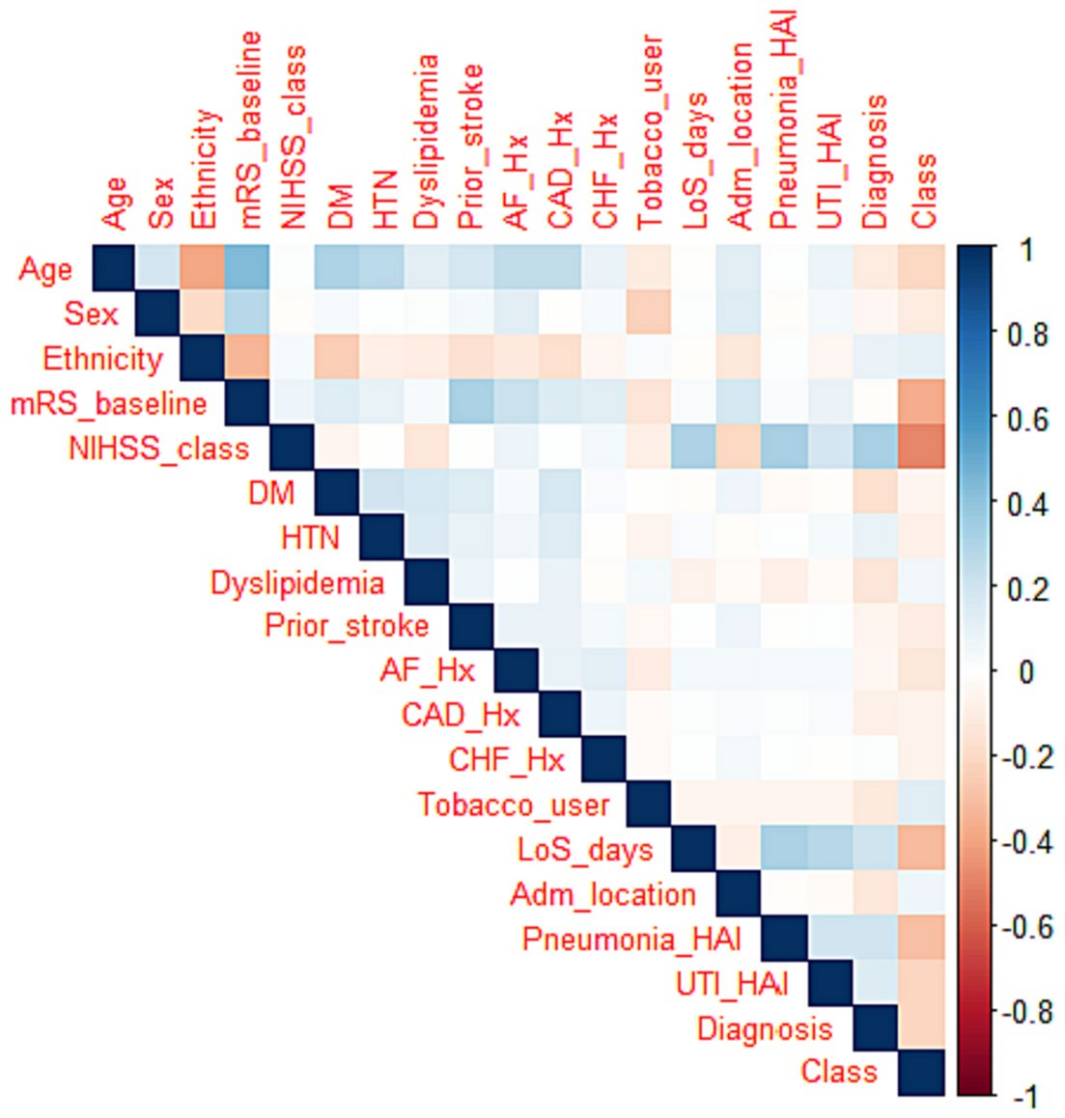
Correlation heat map. Strong positive correlation is represented by blue, strong negative correlation by red, and a lack of color denotes a weak correlation.

Correlation feature selection resulted in three feature categories based on the correlation coefficient. We classify influences as weak, moderate, or strong. Specifically, characteristics with correlation coefficients close to 1 or − 1 are deemed to have a strong positive or strong negative correlation. Furthermore, features with correlation coefficients greater than 0.20 or less than −0.20 are classified as having strong or moderate correlations. See Table 2 and Figure 4.

- Weak Influence: Sex, ethnicity, BMI, DM, HTN, dyslipidemia, prior stroke, AF, CAD, CHF, tobacco use, and admission location.
- Moderate Influence: Age, pneumonia, pre-stroke mRS, LOS, UTI, stroke type.
- Strong Influence: NIHSS.

**Table 2:** The correlation coefficient of each feature with the other features and the class (90 mRS).

Feature	Age	Sex	Ethnicity	mRS_baseline	NIHSS_class
Coefficient	−0.21	−0.11	0.11	−0.36	−0.49
Feature	AF_Hx	CAD_Hx	CHF_Hx	Tobacco_user	LoS_days
Coefficient	−0.12	−0.07	−0.06	0.13	−0.31
Feature	BMI_class	DM	HTN	Dyslipidemia	Prior_stroke
Coefficient	−0.07	−0.05	−0.07	0.05	−0.10
Feature	Adm_location	Pneumonia_HAI	UTI_HAI	Stroke type (Diagnosis)	Class
Coefficient	0.07	−0.29	−0.22	−0.21	1

Based on Permutation/ feature importance, the classifiers ranked variables based on their importance to the prediction accuracy (Figure 3). The cross-analysis resulted in four sets of features (Table 3):

- Strong features with full agreements (SFA4): These features were identified as strong by all four methods, including pneumonia, LOS, stroke type, and NIHSS that has highest correlation coefficient in feature correlation method.
- Strong features with at least three agreements (SFA3): These features were identified as strong by at least three methods, comprising the SFA4 features and adding UTI, dyslipidemia, prior stroke, AF, CAD, CHF, tobacco use, and admission location.
- Weak features (WF12): These features were recognized as strong by only one or two methods, including age, pre-stroke mRS, sex, DM, and HTN.
- Weakest features (WF0): These features were classified as weak by all four feature selection methods, consisting of ethnicity and BMI.

**Table 3:** Cross-analysis of the best features’ qualities of four feature selection methods, (✓) means the feature is selected by the method, and (**×**) otherwise.

Feature	Correlation	RF	ANN	EE
NIHSS at admission	✓**Strong**	✓	**×**	**×**
Age	✓**Moderate**	**×**	**×**	✓
Pneumonia	✓**Moderate**	✓	✓	✓
Pre-stroke mRS	✓**Moderate**	**×**	**×**	**×**
LoS	✓**Moderate**	✓	✓	✓
UTI	✓**Moderate**	**×**	✓	✓
Stroke type	✓**Moderate**	✓	✓	✓
Sex	× Weak	**×**	**×**	✓
Ethnicity	× Weak	**×**	**×**	**×**
BMI	× Weak	**×**	**×**	**×**
DM	× Weak	✓	✓	**×**
HTN	× Weak	✓	✓	**×**
Dyslipidemia	× Weak	✓	✓	✓
Prior stroke	× Weak	✓	✓	✓
AF	× Weak	✓	✓	✓
CAD	× Weak	✓	✓	✓
CHF	× Weak	✓	✓	✓
Tobacco use	× Weak	✓	✓	✓
Admission location	× Weak	✓	✓	✓

### Evaluation of the trained models

**Figure 5:**
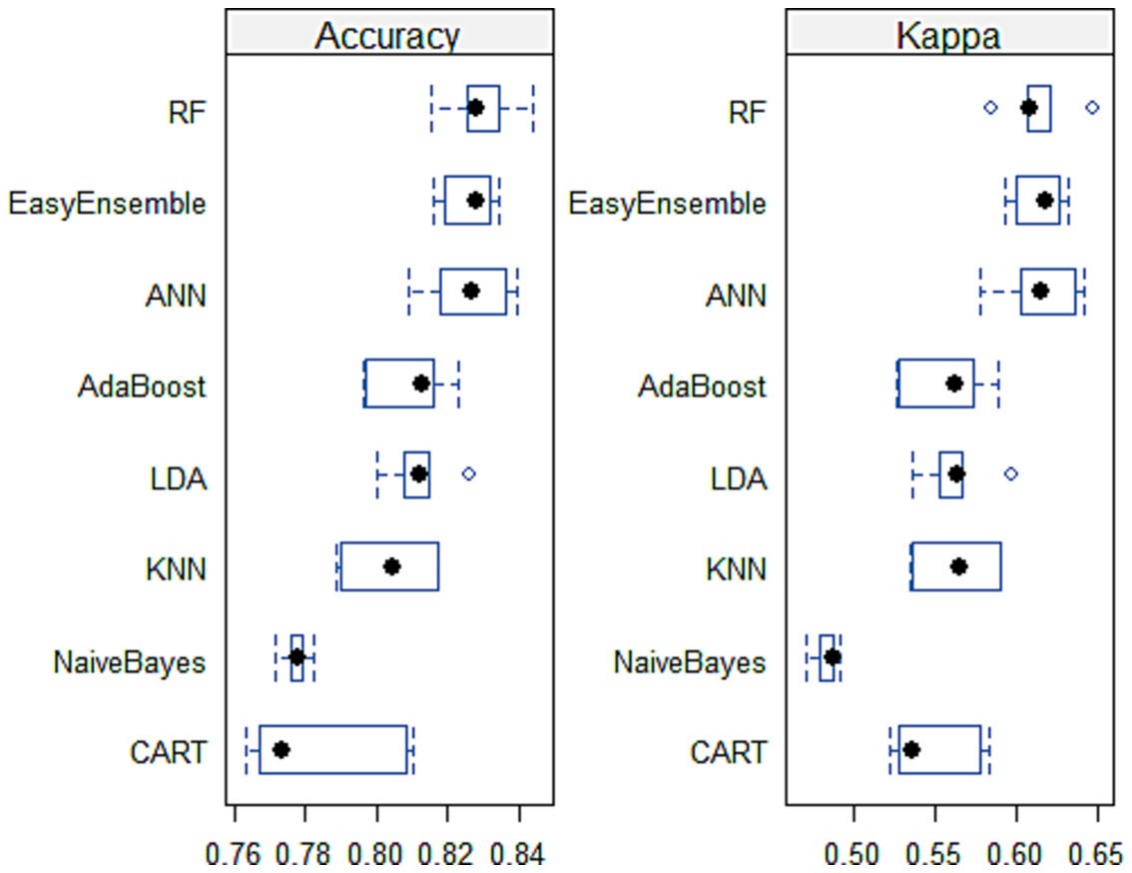
Box and whisker plots for classifiers’ comparison across 5-fold cross-validation.

As demonstrated by Scrutinio and colleagues (42). Choosing the most effective machine learning model is a formidable task. This process demands the consideration of numerous performance parameters while simultaneously weighing the insights derived from the results and their relevance to the clinical field. Therefore, several experiments were conducted on the stroke data to identify the optimal machine learning model for predicting stroke prognosis. Table 4 demonstrates that RF achieved the highest performance, with an average accuracy of 82.9%, consistently showing good results. ANN followed RF in performance. Figure 5 visually confirms RF and ANN’s superiority, as their accuracy boxes are positioned toward the highest values, indicating narrower widths. Additionally, the KAPPA plots show that both classifiers approach 0.65, closer to 1 than other classifiers, signifying excellent consistency and stability over the five runs.

**Table 4:** Accuracy results of different models, using 5-fold cross-validation,

Model	Min.	1stQu.	Median	Mean	3rdQu.	Max.
CART	0.763	0.767	0.773	0.784	0.808	0.810
LDA	0.800	0.808	0.812	0.812	0.815	0.826
SVM	0.803	0.813	0.814	0.819	0.826	0.838
KNN	0.789	0.790	0.805	0.804	0.817	0.818
RF	0.816	0.825	0.828	0.829	0.834	0.844
ANN	0.809	0.818	0.827	0.826	0.836	0.840
NaiveBayes	0.772	0.776	0.778	0.777	0.779	0.782
AdaBoost	0.796	0.797	0.813	0.809	0.816	0.823
EasyEnsemble	0.816	0.819	0.828	0.826	0.832	0.834

**Figure 6:**
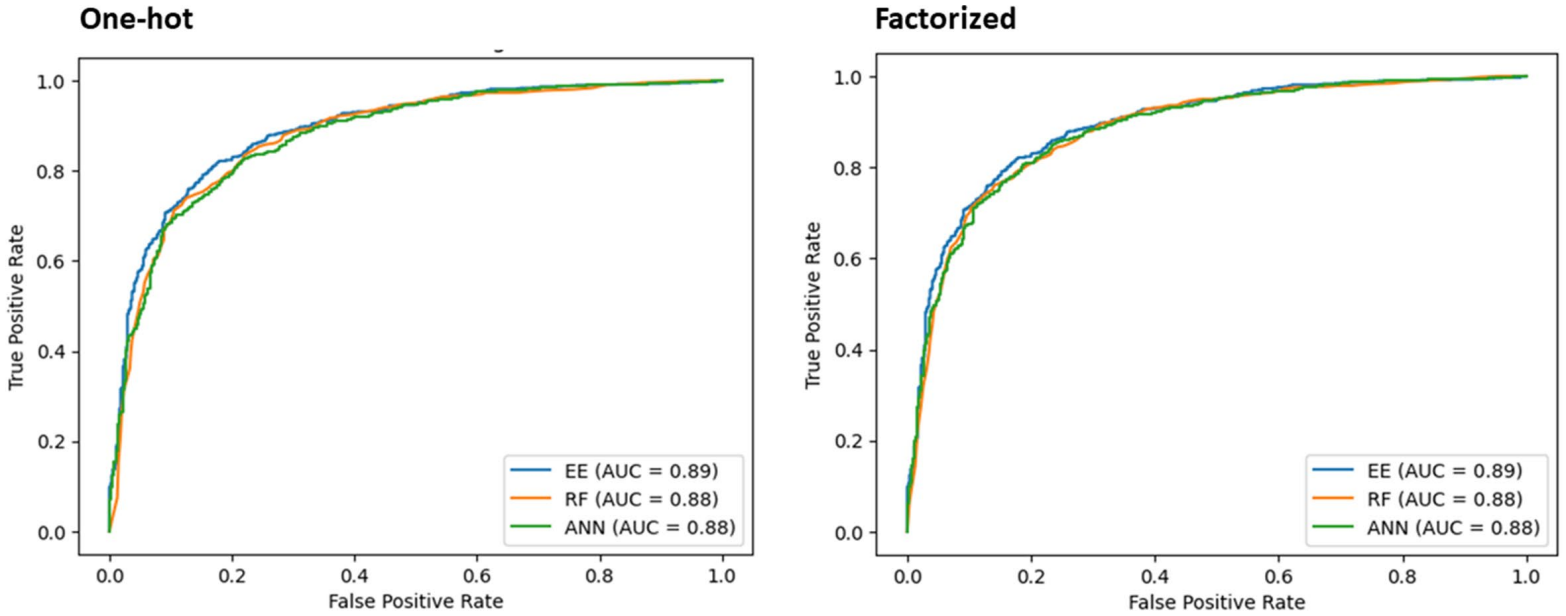
AUC results of the chosen classifiers using one-hot encoding and factorized encoding.

The best performing model, RF, was evaluated using one-hot and factorized encoding. Its performance was compared with ANN in terms of F-score and AUC (Table 5 and Figure 6). Additionally, the findings of the EE classifier were studied and compared to the top performers, RF and ANN, while recording F-score and AUC measures.

**Table 5:** prediction results using 5-fold cross validation,

	Encoding	Average Accuracy	F1	AUC
EE	One-hot	0.816779	0.853147	0.891296
RF		0.819463	0.861981	0.879417
ANN		0.810738	0.857576	0.877465
EE	Factorized	0.816779	0.853147	0.891296
RF		0.817450	0.859938	0.881802
ANN		0.812752	0.859587	0.882489

As displayed in Table 5, the classifiers’ performance using one-hot vs. factorized encoding did not exhibit significant differences. Therefore, factorized method was chosen as it helps maintain a smaller size for the training model. Taking into account the average accuracy, F-score, and AUC combined, RF demonstrated superior performance compared to the other models, making it a suitable choice for deployment.

**Figure 7:**
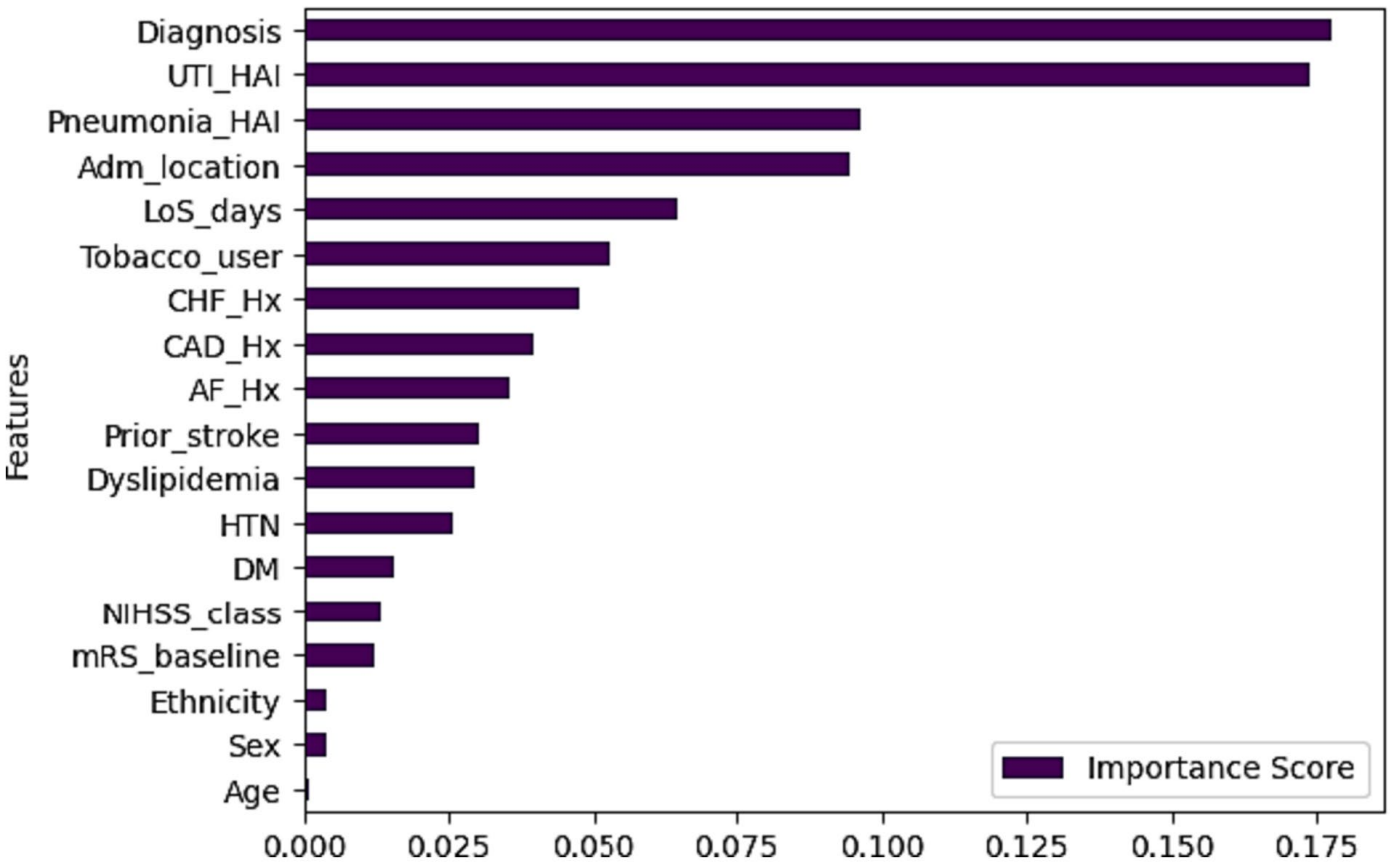
Predictor importance in RF.

In the final phase of this study, a third set of experiments using RF was conducted to explore the features for incorporation into the proposed system (Table 6). Surprisingly, utilizing only the strongest subsets of features (SFA4 and SFA3) hindered the system’s performance. Similarly, when weak and weakest features (WF12&WF0) or the weakest feature (WF0) were removed (as shown in Table 5), most metrics declined, except for a marginal increase in AUC when ethnicity was removed. Eliminating any feature led to a drop in the F1 measure. Based on these findings, the proposed mRS-90 prediction system includes all variables listed in Table 1, except BMI. Table 7 provides a comprehensive overview of the RF model’s performance and Figure 7 presents the significance of predictors.

**Table 6:** The RF prediction results on different subsets of features.

Feature set	TP rate	FP rate	Precision	Recall	F-Measure	AUC
SFA4	0.710	0.340	0.709	0.710	0.709	0.761
SFA3	0.740	0.308	0.739	0.740	0.739	0.797
Removing WF12&WF0	0.765	0.289	0.762	0.765	0.763	0.825
Removing WF0	0.827	0.220	0.825	0.827	0.825	0.893
All features removing the zero BMI records	0.822	0.219	0.821	0.822	0.821	0.892

**Table 7:** RF algorithm performance (the average of 5-folds).

Accuracy	AUC	Sensitivity	Specificity	Precision	Negative predictive value	F1
0.823	0.893	0.88	0.72	0.85	0.77	0.86

The Random Forrest model identified a set of key predictors that help clinicians early predict the 90-day prognosis of stroke patients. The important predictors are illustrated in ascending order (Figure 3) based on their importance on predicting the target outcome.

## Discussion

The use of machine learning (ML) in medical research has expanded significantly, with applications in screening, diagnosis, and prognosis (43). In this study, we aimed to devise a predictive tool based on ML to help clinicians forecast the 90-day prognosis of stroke patients after hospital discharge. Several algorithms were compared for predictive performance, and the RF algorithm emerged as the best performing classifier. Consequently, the discussion section focuses on the insights derived from the RF algorithm’s output.

Two key findings emerged from this study; firstly, feature selection revealed several variables with significant predictive power for the 90-day prognosis, including stroke type, hospital-acquired infections (UTI and pneumonia), admission location, known comorbidities, and other risk factors such as smoking. Secondly, the discrimination power (AUC) of all the models was excellent (0.893) (44), providing a high degree of confidence in the accuracy of the model’s predictions. Also, the RF model’s performance surpassed conventional statistical methods heavily relying on logistic regression to predict disease outcomes, where AUC is typically <0.8 (11, 45, 46).

Past studies have shown that predicting the 90-day prognosis is influenced by factors such as stroke severity, sex, age, stroke type, mRS and NIHSS scores at admission and discharge, body mass index (BMI), comorbidities, smoking history, and in-hospital length of stay (9, 11, 12, 14). In this study, we identified several predictors and ranked them based on their importance, as presented in Figure 7. Predictor importance is determined by the variable’s impact on the model’s ability to predict the outcome, with the most influential predictor ranked first. Subsequently, other variables are ranked relative to the most influential predictor (47).

This study identified the stroke type as the strongest predictor for the 90-day prognosis. In our secondary analysis, we found that 59% of patients with ICH had unfavorable mRS at the 90-day follow-up, while only 31.5% of patients with IS had unfavorable outcomes (value of *p* < 0.05). This indicates that ICH serves as an early predictor of an unfavorable prognosis, aligning with previous research that associates ICH with adverse outcomes, particularly mortality, when compared to IS (48). Furthermore, the severity of presentation at the hospital was higher for patients diagnosed with ICH. Only 8.9% of IS patients presented with an NIHSS score greater than 16, whereas 39.8% of ICH patients had a higher severity score (value of *p* < 0.05). Moreover, the occurrence of hospital-acquired UTI and pneumonia emerged as strong predictors for stroke patient prognosis. Stroke patients are known to be at significant risk of infections during hospitalization, which can worsen their functional outcomes (49). The study revealed that 88.1% of patients who experienced hospital-acquired UTI and 90% of those who developed hospital-acquired pneumonia had an unfavorable 90-day mRS (value of *p* < 0.05).

Admission location significantly influenced the 90-day prognosis. Among patients admitted to the critical care unit, 77.9% had an unfavorable prognosis, contrasting with 33.8 and 26.3% in the stroke unit and other units, respectively (value of *p* < 0.05). This relationship might be associated with factors like the severity of presentation, leading to critical care unit admissions. 64% of critical care unit patients had NIHSS score > 16 compared to 8.3 and 5.8% in the stroke unit and general scope units, respectively (value of *p* < 0.05). Additionally, a majority of critical care unit patients were diagnosed with ICH (61.6% vs. 38.4%), explaining the worse prognosis compared to other admission locations. The literature supports that specialized stroke units lead to favorable outcomes (50, 51), but patients in critical care units tend to have poorer prognoses (52).

Similar to previous research, the LOS was significantly correlated with the 90-day mRS (12). Patients with unfavorable 90-day mRS had an average LOS of 10.3 days, compared to 4.4 days for those with favorable outcomes (value of *p* < 0.05).

The study revealed that smoking history plays a role in predicting the 90-day prognosis. Surprisingly, individuals with a history of tobacco consumption had a more favorable prognosis compared to non-smokers. Only 24.1% of tobacco users had an unfavorable 90-day mRS, while 39.4% of non-smokers had unfavorable outcomes (value of *p* < 0.05). This interesting phenomenon is known as the tobacco paradox in stroke, where smokers exhibit more favorable outcomes than non-smokers (53). Some researchers attribute this paradox to the age difference between the smoker and non-smoker groups. In other words, “the more you smoke, the earlier you stroke and the longer you have to cope” (53). Consistent with previous research, this study found that tobacco consumers had a mean age of 51, whereas non-smokers had a mean age of 55 (value of *p* < 0.05). This finding shed light on the complex relationship between smoking history, age, and stroke prognosis.

Regarding comorbidities, the study found that, apart from dyslipidemia, patients with CHF, CAD, AF, prior stroke, HTN, and DM were more prone to poorer 90-day prognosis (69.6% vs. 35.9, 45% vs. 35, 60.7% vs. 34.8, 49.5% vs. 34.5, 38.2% vs. 30.4, 38.5% vs. 33.5% respectively) with a value of *p* < 0.05. These findings align with much of the past research (11, 14, 48). Dyslipidemia, known as a major risk factor for developing stroke and affecting stroke outcomes (54), showed an interesting result in this study. Patients with dyslipidemia were found to be less prone to unfavorable 90-day mRS, with 33.4% compared to 38.4% for non-dyslipidemia patients (value of *p* < 0.05). In a meta-analysis that studied the impact of several comorbidities on stroke outcomes, particularly recurrence, dyslipidemia turned to have insignificant relationship (55). It’s important to consider the specific characteristics and interactions within the study population when interpreting the impact of dyslipidemia on stroke outcomes.

Consistent with existing literature, the severity of stroke, as measured by NIHSS, is a strong predictor of stroke outcomes and prognosis (9, 11, 14). This study corroborates these findings, revealing that higher admission NIHSS scores are associated with an elevated risk of unfavorable 90-day mRS. Specifically, 85.4% of patients with severe NIHSS scores (>16) had unfavorable 90-day mRS, in contrast to 27.5% of those with NIHSS scores <16 (value of *p* < 0.05). Furthermore, the mRS prior to stroke onset, collected from family members and calculated by treating physicians, plays a significant role in predicting prognosis. Patients with mRS > 2 before the latest stroke onset were more likely to have an unfavorable 90-day mRS. Notably, 92.8% of patients with mRS > 2 prior to stroke onset had unfavorable mRS at 90 days, compared to 38.5% of those with mRS ≤ 2 (10, 12).

Ethnicity also imposes a significant risk of stroke development among certain groups (56). In this study, patients belonging to the MENA region had a 42.5% risk of unfavorable 90-day mRS, whereas patients from South-Asia, South-East Asia, and other ethnicities had percentages of 32.3, 33.9, and 30.6%, respectively, (value of *p* < 0.05). Interestingly, Qatari patients had the highest risk of unfavorable 90-day mRS compared to all other patients, with 48.7% versus 33.3% (value of *p* < 0.05). This observation may be attributed to Qatar’s unique demographic structure, where the majority of the population comprises, expatriates living and working in Qatar (4, 24). Consequently, they tend to be significantly younger than Qatari patients at the time of stroke presentation, with a mean age of 64 ± 14 years compared to 52.8 ± 12 years (value of *p* < 0.05).

Patient sex plays a significant role in predicting stroke prognosis (9, 12). This study found that 46.3% of female patients had unfavorable 90-day mRS, compared to 33.5% of male patients (value of *p* < 0.05). Although there was no significant difference in stroke severity between male and female patients, the mean age of female patients was significantly higher than that of male patients (59.5 ± 16 years vs. 53 ± 13 years, value of *p* < 0.05). This finding is consistent with previous research conducted in Qatar (57). Age, as in previous studies, was found to significantly predict stroke prognosis (11). The study found that the average age of stroke patients with unfavorable 90-day mRS was significantly higher than the average age of those with a favorable mRS; 58 ± 15 years vs. 52 ± 12 years (value of *p* < 0.05). This further emphasizes the importance of considering age as a relevant factor in early predicting stroke outcomes.

Body Mass Index (BMI) was excluded in the model training and testing phase as it ranked as one of the weakest features (WF0) and was not prioritized by any feature selection methods. Including BMI led to a significant decline in system performance. Additionally, around 5% of included records had missing BMI values, and training models on approximate or hypothesized data in the medical field can distort predictive performance, impacting clinical decision-making (58, 59). Thus, BMI was not considered in the final predictive model. Another option is to exclude all the instances where the BMI is missing. Here we eliminated 393 instances with BMI = 0 from the dataset. We found that the predictive performance, as shown in Table 6, was marginally lower than when the full feature was excluded. We acknowledge the significance of quantifying the influence of data handling decisions on model performance, and we have recorded these findings to demonstrate the trade-off between removing the feature that has missing data and preserving predictive accuracy. However, this could be a dilemma: which to remove? the missing data instances? Or the entire feature? The first option eliminates a number of instances that may be significant to the learning process, whereas the second option eliminates a feature that may be important to the learning process. As a result, we believe that answering such a question is primarily dependent on the dataset investigated; in our dataset, we found that it is preferable to delete the entire feature from the final prediction system.

From another angle, this study demonstrates that the Random Forest (RF) model outperformed the other tested models, achieving maximum accuracy and excellent discrimination power (AUC > 0.8). This promising finding suggests that the RF model can be deployed in clinical settings to early predict the 90-day prognosis, enabling care providers to devise personalized plans that enhance preventive measures and ensure better quality of life for stroke patients.

The RF model demonstrated superior performance compared to conventional statistical methods, which often rely on logistic regression with AUCs typically <0.8 (11, 45, 46). The powerful predictive capacity of machine learning makes prospective validation of the RF algorithm essential to assess its real-world performance in clinical settings (60). External validation remains a critical step for clinical implementation of recent machine learning models. This study lays the groundwork for further validation and potential real-world deployment of the RF model, opening new avenues for stroke prognosis prediction and patient care. Future prospective deployment and validation should prioritize high-quality, well-described databases with sufficient sample sizes, comprehensive patient tracking, and clinically significant endpoints.

## Limitations

The study has several limitations that may present opportunities for future research in this field. The study’s predictive model demonstrated impressive performance in predicting stroke outcomes, providing valuable insights into prognosis prediction. However, to ensure the reliability and applicability of the model, external validation in diverse patient populations is crucial. This external validation will test the model’s robustness and verify its effectiveness across different healthcare settings and stroke cases.

Data quality issues led to the exclusion of certain variables from the prediction system. While this was necessary to maintain data integrity, it is important to recognize that excluding BMI, that has been found in previous research to play a significant role in predicting stroke outcomes. Exploring ways to address data quality and incorporate important variables like BMI in future studies may enhance the model’s predictive capabilities.

Moreover, the study’s context in Qatar might limit its generalizability to other regions with different demographic and healthcare characteristics. To increase the model’s applicability worldwide, similar studies should be conducted in diverse populations, considering regional variations in stroke risk factors and healthcare practices.

In summary, despite its promising performance, the study’s predictive model needs further validation, inclusion of imaging data, and consideration of variables excluded due to data quality issues to ensure its effectiveness and applicability in diverse clinical settings and populations. Addressing these limitations will contribute to the advancement of stroke prognosis prediction, ultimately leading to improved patient care and outcomes.

## Conclusion

The results of this study highlight the superiority of the Random Forest (RF) model over other tested models, showcasing its remarkable accuracy and discrimination power (AUC 0.893). This promising finding opens avenues for deploying the RF model in clinical settings to early predict 90-day prognosis, enabling personalized care plans that enhance preventive measures and improve the quality of life for stroke patients.

The RF model’s performance surpasses conventional statistical methods, such as logistic regression, commonly yielding lower AUC values. Embracing the powerful predictive capacity of machine learning, it is imperative to prospectively validate the RF algorithm to assess its real-world clinical performance. This validation process will provide essential insights into the model’s effectiveness, reliability, and potential for practical implementation in clinical settings. By rigorously examining its predictive capabilities, full potential of the RF model can be unlocked, advancing stroke prognosis prediction and enhancing patient care outcomes.

The study lays the foundation for further validation and potential real-world deployment of the RF model, representing a significant step forward in stroke prognosis prediction and patient care. By embracing machine learning predictive capacity, healthcare providers can better tailor interventions and optimize outcomes for stroke patients, ultimately advancing the field of stroke research and treatment.

## Data availability statement

The raw data supporting the conclusions of this article will be made available by the authors, without undue reservation.

## Ethics statement

The studies involving humans were approved by the Institutional Review Board (IRB) at the Medical Research Center, Hamad Medical Corporation, Doha, Qatar (MRC-01-22-594). The studies were conducted in accordance with the local legislation and institutional requirements. Written informed consent for participation was not required from the participants or the participants’ legal guardians/next of kin in accordance with the national legislation and institutional requirements.

## Author contributions

AA: Conceptualization, Data curation, Formal analysis, Methodology, Writing – original draft, Writing – review & editing. IA: Data curation, Formal analysis, Methodology, Writing – original draft, Writing – review & editing. YI: Conceptualization, Data curation, Methodology, Writing – original draft, Writing – review & editing. AN: Data curation, Methodology, Writing – original draft, Writing – review & editing. NA: Data curation, Methodology, Writing – original draft, Writing – review & editing. OA: Data curation, Methodology, Writing – original draft, Writing – review & editing. AT: Data curation, Formal analysis, Methodology, Writing – original draft, Writing – review & editing. AH: Data curation, Formal analysis, Methodology, Writing – original draft, Writing – review & editing.
