# Corrigendum: Predicting 90-day prognosis for patients with stroke: a machine learning approach

**DOI:** 10.3389/fneur.2024.1470237

**Published:** 2024-08-07

**Authors:** Ahmad A. Abujaber, Ibraheem M. Alkhawaldeh, Yahia Imam, Abdulqadir J. Nashwan, Naveed Akhtar, Ahmed Own, Ahmad S. Tarawneh, Ahmad B. Hassanat

**Affiliations:** ^1^Nursing Department, Hamad Medical Corporation (HMC), Doha, Qatar; ^2^Faculty of Medicine, Mutah University, Al-Karak, Jordan; ^3^Neurology Section, Neuroscience Institute, Hamad Medical Corporation (HMC), Doha, Qatar; ^4^Neuroradiology Department, Neuroscience Institute, Hamad Medical Corporation (HMC), Doha, Qatar; ^5^Faculty of Information Technology, Mutah University, Al-Karak, Jordan

**Keywords:** stroke, prognosis, ischemic stroke, hemorrhagic stroke, machine learning

In the published article, there was an error in the Funding statement, which previously stated: The author(s) declare financial support was received for the research, authorship, and/or publication of this article. Open Access funding provided by the Qatar National Library. The correct Funding statement appears below.

